# Stem-cell-specific endocytic degradation defects lead to intestinal dysplasia in *Drosophila*

**DOI:** 10.1242/dmm.023416

**Published:** 2016-05-01

**Authors:** Péter Nagy, Laura Kovács, Gyöngyvér O. Sándor, Gábor Juhász

**Affiliations:** 1 Department of Anatomy, Cell and Developmental Biology, Eötvös Loránd University, Pázmány s. 1/C, Budapest H-1117, Hungary; 2 Institute of Genetics, Biological Research Centre of the Hungarian Academy of Sciences, Temesvári krt. 62, Szeged H-6726, Hungary

**Keywords:** Autophagy, *Drosophila*, Endocytosis, UVRAG

## Abstract

UV radiation resistance-associated gene (UVRAG) is a tumor suppressor involved in autophagy, endocytosis and DNA damage repair, but how its loss contributes to colorectal cancer is poorly understood. Here, we show that UVRAG deficiency in *Drosophila* intestinal stem cells leads to uncontrolled proliferation and impaired differentiation without preventing autophagy. As a result, affected animals suffer from gut dysfunction and short lifespan. Dysplasia upon loss of UVRAG is characterized by the accumulation of endocytosed ligands and sustained activation of STAT and JNK signaling, and attenuation of these pathways suppresses stem cell hyperproliferation. Importantly, the inhibition of early (dynamin-dependent) or late (Rab7-dependent) steps of endocytosis in intestinal stem cells also induces hyperproliferation and dysplasia. Our data raise the possibility that endocytic, but not autophagic, defects contribute to UVRAG-deficient colorectal cancer development in humans.

## Abstract (precis)

***Drosophila* Collection:** Intestinal-stem-cell-specific loss of the *Drosophila* ortholog of the tumor suppressor UVRAG, which is implicated in colorectal cancer, leads to endocytic defects and dysplasia.

## INTRODUCTION

UV radiation resistance-associated gene (*UVRAG*) encodes a subunit of the Vps34 kinase complex, which is important for the generation of phosphatidylinositol 3-phosphate (PI3P). UVRAG promotes the biogenesis of this phospholipid by binding to the autophagic tumor suppressor Beclin-1 within this complex (Itakura et al., 2008; Liang et al., 2006). PI3P is enriched not only on endosomes but also on early autophagic structures, including phagophores and autophagosomes, and it mediates the recruitment of downstream effectors involved in autophagosome and endosome maturation (Kutateladze, 2012). UVRAG is also reported to bind to HOPS (Liang et al., 2008), a tethering complex that promotes multiple trafficking routes to lysosomes, including autophagy, endocytosis, and the biosynthetic transport of hydrolytic enzymes and membrane proteins. In cancer cell lines, overexpression of *UVRAG* is found to promote autophagy and reduce cell proliferation, raising the possibility that its tumor suppressor function involves the regulation of autophagy (Liang et al., 2006). In line with that possibility, transposon-induced *Uvrag*-mutant mice display impaired autophagic degradation in cardiomyocytes and fibroblasts (Song et al., 2014). However, conflicting reports have also been recently published that have found no crucial role for UVRAG in autophagosome formation and fusion with lysosomes (Jiang et al., 2014; Takáts et al., 2014). A potential explanation for these discrepancies is cell-type-specific differences in UVRAG function.

It is worth noting that UVRAG might also act independently of its roles in vesicular trafficking. For example, it contributes to the maintenance of chromosomal stability by promoting repair after DNA damage (Zhao et al., 2012). In fact, UVRAG was originally discovered as a factor that partially complements the sensitivity to UV of xeroderma pigmentosum group C cells (Teitz et al., 1990). Thus, UVRAG function must be studied in the proper cellular and developmental context in order to understand its tissue-specific roles.

Progression of solid tumors is accelerated by genome instability owing to either chromosome instability or microsatellite instability. In the latter case, dysfunction of the mismatch repair pathway leads to somatic mutations in genes containing repetitive DNA sequences. One such gene is *UVRAG*, which contains an A10 exonic repeat. Mutations in *UVRAG* have indeed been found in colorectal cancer cells with microsatellite instability, indicating that the protein could function as a tumor suppressor in humans (Ionov et al., 2004). As a consequence, *UVRAG* loss has been suggested to contribute to the development of colorectal cancer, but there is still no experimental support for the *in vivo* relevance of this model.

There are remarkable similarities between the cell types and signaling pathways that are important for fly and mammalian gut physiology (Jiang and Edgar, 2012). For example, proliferation of intestinal stem cells (ISCs) in *Drosophila* ensures self renewal and generates progenitor cells called enteroblasts (EBs) that produce enterocytes and enteroendocrine cells, similar to the functions of stem cells residing at the base of Lieberkühn's crypts in the mammalian intestine. Ingested pathogens and toxins damage the gut and trigger a regeneration response through increased proliferation of stem cells and differentiation of progeny, both in flies and mammals (Jiang and Edgar, 2012). We thus decided to analyze whether the role of UVRAG as a tumor suppressor is evolutionarily conserved in the adult *Drosophila* intestine and to understand which of its diverse functions might be relevant in a setting similar to that during colorectal cancer development.

## RESULTS

### UVRAG is important for endosome maturation in ISCs

**Fig. 1.**
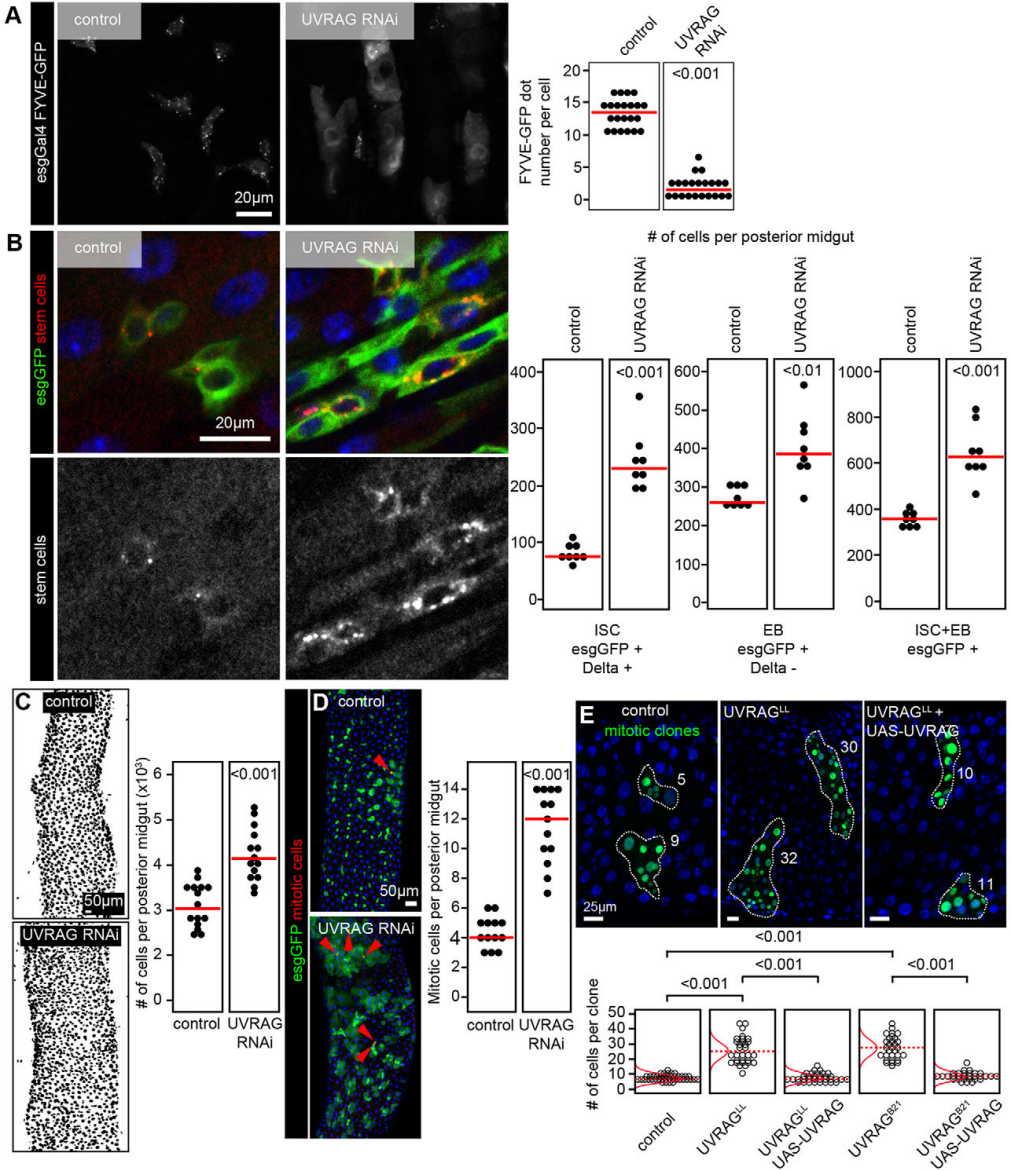
**ISC-specific loss of UVRAG leads to dysplasia.** (A) Silencing of *Uvrag* in esg-GFP-positive stem and progenitor cells impairs PI3P-associated FYVE-GFP puncta formation. Each full circle in the graphs shown on the right represents the number of FYVE-GFP dots per cell. (B) RNAi knockdown of *Uvrag* in esg-GFP-positive cells increases the number of Delta-positive ISCs and Delta-negative EBs in the posterior midgut of 3-week-old adult flies. Note the large-scale accumulation of Delta in intracellular compartments of UVRAG RNAi cells. Each full circle represents the number of cells per posterior midgut of a single animal in these and all subsequent charts. (C) Loss of UVRAG in stem and progenitor cells increases overall cell number in the posterior midgut. (D) Silencing of *Uvrag* induces the proliferation of ISCs, based on the elevated number of mitotic (phosphorylated-histone-H3-positive, arrowheads) cells. (E) Positively marked (GFP positive) mitotic clones of *Uvrag*^LL^ null mutant cells contain more cells than control clones two weeks after clone induction, which is suppressed by transgenic expression of wild-type *Uvrag*. The quantification shows data from this panel as well as Fig. S1F, showing that mitotic clones for the independent null mutation *Uvrag*^B21^ produces the same phenotypes. Red lines indicate median in A-D, and distribution of data points in E, with dotted red lines indicating median. Because experiments were carried out in parallel, the control and UVRAG RNAi data in D is the same as that shown in Fig. 5C,D, and the control data in B,D is the same as in Fig. 6G.

*UVRAG* mutations arise from microsatellite instability in human colorectal cancers. To understand the consequences of the adult-onset loss of this gene, we induced RNA interference (RNAi)-mediated silencing of *Uvrag* in midgut ISCs of adult *Drosophila* using a standard temperature-sensitive gene expression system. This method allows genetic manipulation of escargot (esg)-positive ISCs and differentiating progenitors (EBs) in adult flies, as gene silencing (or overexpression) and GFP expression can be triggered by shifting animals to 29°C (Micchelli and Perrimon, 2006). Knockdown of *Uvrag* in esg-GFP-positive cells (where GFP is expressed under the *esg* promoter) strongly decreased the number of GFP-tagged FYVE dots, which mark PI3P-positive vesicles, indicating efficient gene silencing (Fig. 1A).

Loss of UVRAG results in the accumulation of plasma membrane receptors and ligands in stalled endocytic compartments in cultured human cells, and also in cells of the developing *Drosophila* eye and wing, which might interfere with the activity of various signaling pathways (Jiang et al., 2014; Lee et al., 2011; Lőrincz et al., 2014). During ISC proliferation and differentiation, Notch receptor and its ligand Delta traffic via endosomes (Montagne and Gonzalez-Gaitan, 2014). In line with this, RNAi against *Uvrag* (UVRAG RNAi) in esg-GFP-positive cells resulted in a striking intracellular accumulation of the Notch ligand Delta (Fig. 1B; Fig. S1A). To confirm this finding, we generated mitotic clones in the gut that were homozygous mutants for previously described *Uvrag*-null alleles (Lee et al., 2011; Takáts et al., 2014). Both mutations in *Uvrag* also resulted in intracellular Delta accumulation (Fig. S1B), in line with our RNAi data. Basal levels of the Wnt signaling ligand Wingless/Wg can be detected in ISCs and EBs (Cordero et al., 2012; Lin et al., 2008), and the loss of *Uvrag* results in increased punctate intracellular Wg signals (Fig. S1C), again indicating impaired endolysosomal degradation.

UVRAG- or Vps34-dependent production of the phospholipid PI3P is important for endosome maturation. We thus analyzed Rab7-positive endosomes in control and UVRAG loss-of-function stem cells. Indeed, quantification of Rab7-GFP vesicles revealed a clear increase in the percentage of cytoplasmic area occupied in UVRAG-deficient cells (Fig. S1D).

### UVRAG deficiency leads to intestinal dysplasia

Strikingly, the loss of UVRAG led to a remarkable expansion of both ISCs containing the endocytosed Notch ligand Delta and Delta-negative EBs (Fig. 1B). The overall cell number in the posterior midgut of these animals also increased (Fig. 1C), as well as the thickness of the intestinal wall (Fig. S1E).

Staining for the mitotic marker phosphorylated histone H3 revealed that ISCs proliferated more upon loss of UVRAG (Fig. 1D). This was further confirmed with the help of genetic mosaics in which *Uvrag*-mutant clones were induced by somatic recombination at the adult stage: GFP-positive ISCs that were homozygous mutant for either one of the two null alleles produced many more cells per clone than control cells during the same time window (Fig. 1E; Fig. S1F). Importantly, the expansion of *Uvrag*-mutant clones was suppressed by expression of wild-type *Uvrag* in these cells in both cases (Fig. 1E; Fig. S1F), confirming that the intestinal dysplasia is solely due to UVRAG deficiency. *Uvrag* overexpression on its own caused no obvious alteration of the esg-GFP compartment in the posterior midgut (Fig. S1G).

### Loss of UVRAG causes differentiation defects and cell shape changes

**Fig. 2.**
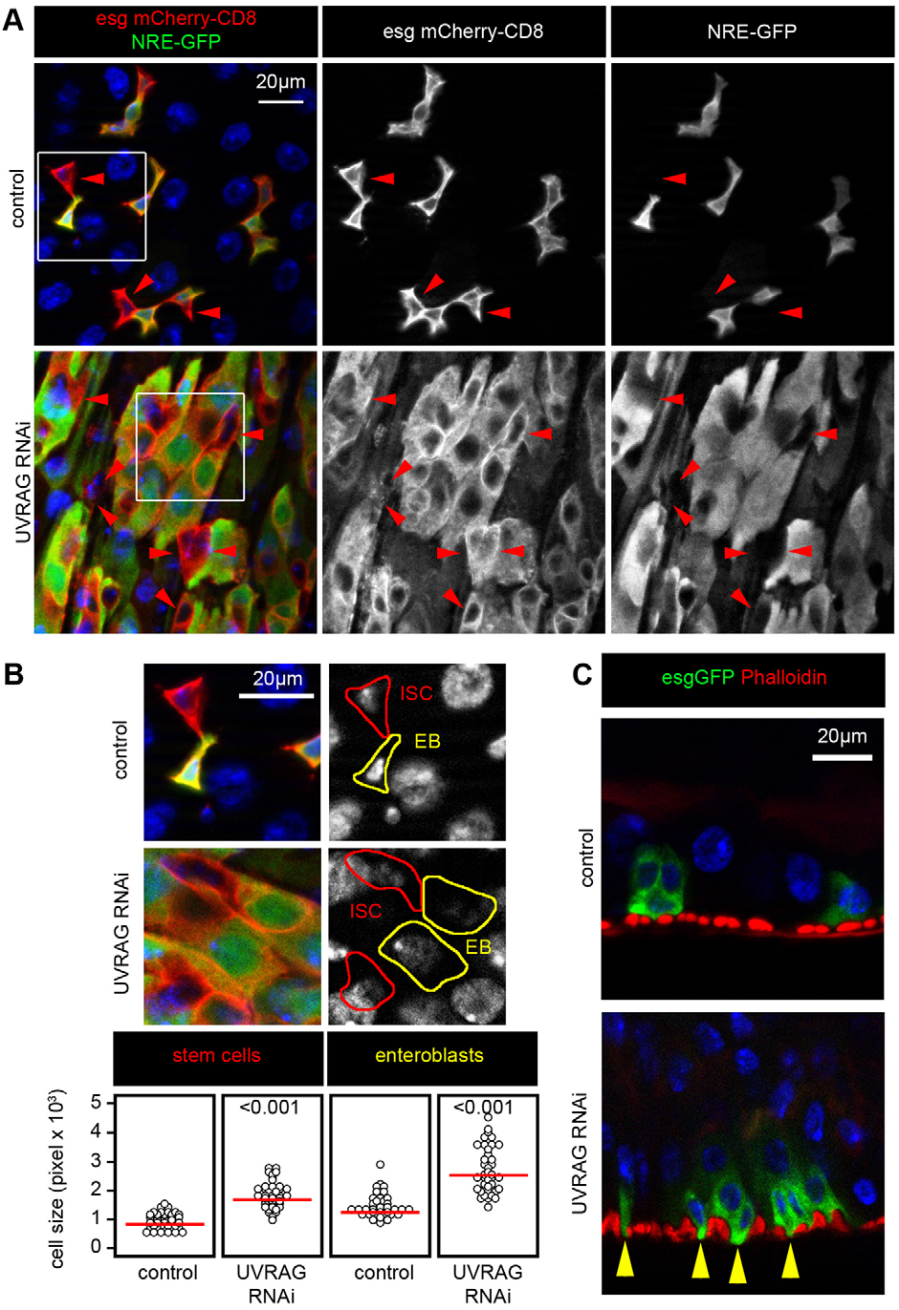
**UVRAG deficiency impairs ISC differentiation and shape.** (A) The expression of the Notch activity reporter NRE-GFP, which expresses GFP under the control of a Notch response element, is restricted to esg-mCherry-positive EBs in control animals, but it remains active in the large polyploid cells seen in *Uvrag*-knockdown intestines. Red arrowheads highlight ISCs, which are esg-mCherry positive and NRE-GFP negative. Boxed areas are shown enlarged in B. (B) UVRAG RNAi increases the size of both ISCs and EBs. Note that the shape of these cells is always round, unlike in control intestines. (C) UVRAG-deficient cells grow cytoplasmic protrusions (yellow arrowheads) toward the underlying muscle cell layer.

ISCs have the ability to self renew and to generate EB daughter cells in which Notch signaling is activated by ISCs that are positive for its ligand Delta (Ohlstein and Spradling, 2007). In wild-type guts, most EBs differentiate into enterocytes. EBs become polyploid and downregulate both esg expression and Notch signaling during this differentiation process. In contrast, UVRAG-deficient cells retained both *esg-Gal4*-driven UAS-mCherry (esg-mCherry) expression and active Notch signaling even after becoming polyploid (Fig. 2A). Moreover, the esg-GFP-positive cells with UVRAG RNAi failed to express the enterocyte differentiation marker Myo1A (Fig. S2A). The activity of target of rapamycin (Tor) kinase is relatively low in ISCs, which maintains their stem cell identity. Notch-mediated activation of this important positive regulator of cell growth is reported to promote the differentiation of EBs into enterocytes (Kapuria et al., 2012). In line with the impaired differentiation of UVRAG RNAi cells, all esg-GFP-positive cells showed lower Tor activity than surrounding enterocytes based on the phosphorylation levels of its substrate 4EBP (Fig. S2B).

In addition, the size of both Delta-negative EBs and Delta-positive ISCs was increased by the loss of UVRAG (Fig. 2B), and cytoplasmic protrusions that extended towards the underlying muscle layer appeared on these cells (Fig. 2C). The number of enteroendocrine cells was unchanged in UVRAG-deficient intestines (Fig. S2C). As these cells represent another lineage that also differentiates from EBs (Jiang and Edgar, 2012), this result indicates that UVRAG loss specifically leads to the accumulation of EBs that are committed to an EC fate.

### UVRAG is required for gut function and survival during normal conditions and in response to stress

**Fig. 3.**
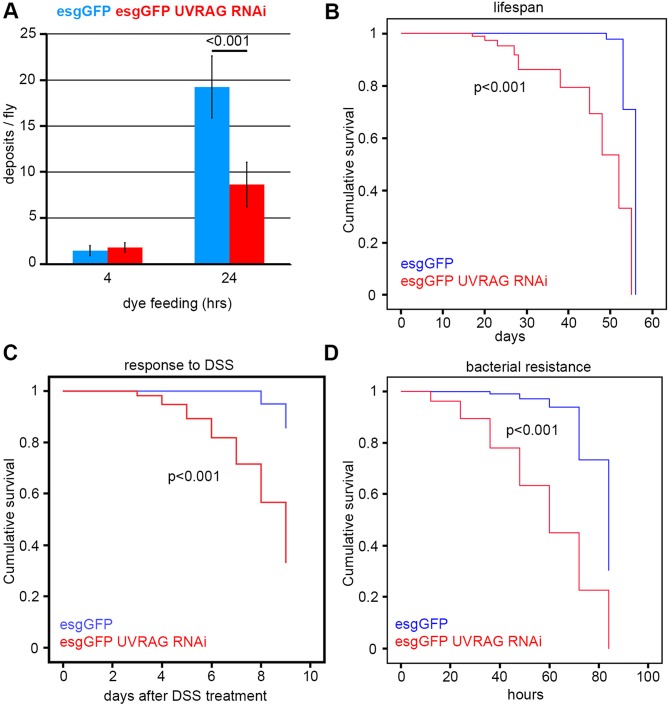
**UVRAG loss impairs gut function and survival.** (A) Stem- and progenitor-cell-specific knockdown of *Uvrag* in the intestine leads to increased retention of ingested Bromophenol-Blue-containing food (a constipation-like phenotype). Error bars represent s.d. (B) Loss of UVRAG in ISCs and EBs decreases lifespan. (C) Treatment with the toxin DSS kills UVRAG RNAi flies much faster than control flies. (D) Animals undergoing *Uvrag* knockdown in esg-positive cells are hypersensitive to infection by *Pseudomonas aeruginosa*.

To understand the physiological relevance of intestinal dysplasia caused by the lack of UVRAG, we analyzed gut function in wild-type and esg-specific loss-of-function animals. Feeding flies a blue-dye-containing food revealed that animals with dysplastic guts defecated significantly less 24 h after starting the experiments (Fig. 3A), even though their food intake was similar to that of controls (Fig. S3A), indicating increased retention of ingested food. As proper gut function is a crucial determinant of *Drosophila* lifespan (Biteau et al., 2010), we next followed the long-term survival of control flies and those with knockdown of *Uvrag* in ISCs and EBs. These experiments revealed an obvious reduction of lifespan in the animals with UVRAG-deficient dysplastic guts (Fig. 3B).

We hypothesized that intestinal dysplasia sensitizes the flies to stressors that target the intestine. A common model for experimental colitis is to feed *Drosophila* or mice with the toxin dextran sodium sulfate (DSS) (Amcheslavsky et al., 2009). DSS treatment induces a regeneration response that is characterized by large-scale ISC proliferation and subsequent differentiation in wild-type guts; however, DSS treatment did not further enhance cell proliferation in dysplastic UVRAG-deficient guts (Fig. S3B). In line with this, DSS killed flies undergoing UVRAG RNAi in esg-GFP-positive cells much faster than it killed control flies (Fig. 3C).

Another commonly used experimental stressor of the gut is bacterial infection (Lemaitre and Miguel-Aliaga, 2013). Indeed, UVRAG loss-of-function animals were highly sensitive to oral administration of the pathogen *Pseudomonas aeruginosa* (Fig. 3D).

### UVRAG regulates STAT and JNK signaling in the gut

We tested the activity of selected signaling pathways in UVRAG-deficient intestines that could play a crucial role in the proliferation of ISCs of the posterior midgut (Jiang and Edgar, 2012; Lemaitre and Miguel-Aliaga, 2013). Receptor tyrosine kinases such as epidermal growth factor receptor (Egfr) and insulin receptor (InR) signal through the Ras-MAPK cascade and Akt, respectively. We could not detect increased activation of these pathways in UVRAG RNAi cells, based on similar staining intensities using antibodies specific for the active and phosphorylated forms of MAPK and Akt, respectively (Fig. S4A,B).

**Fig. 4.**
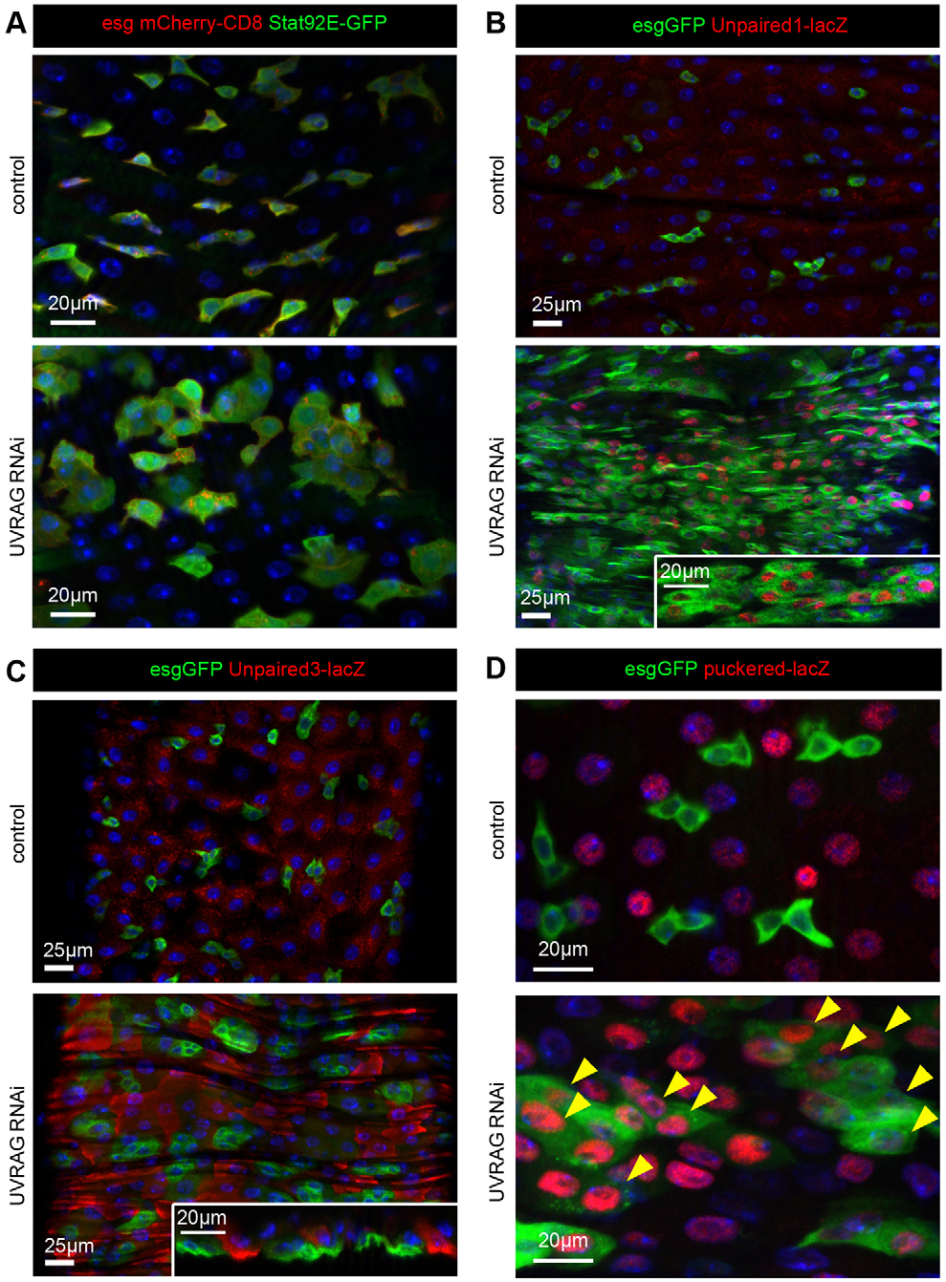
**STAT and JNK signaling are active in UVRAG-deficient cells.** (A) The JAK-STAT activity reporter Stat92E-GFP is active in all esg-mCherry-positive UVRAG RNAi cells. (B) Loss of UVRAG strongly increases the expression of the cytokine reporter *upd1-lacZ* compared to control guts. The majority of esg-GFP-positive cells undergoing UVRAG RNAi are positive for the nuclear *lacZ* reporter. (C) Expression of the *upd3-lacZ* reporter is increased in enterocytes surrounding esg-GFP-positive UVRAG RNAi cells compared to control guts of 3-week-old animals. (D) The JNK activity reporter *puc-lacZ* is activated in the majority of esg-GFP-positive *Uvrag*-knockdown cells (arrowheads), in addition to its normal expression in enterocytes. Note that β-gal, as expressed from *lacZ*, is in the nucleus owing to a nuclear localization signal.

Increased cytokine signaling through the JAK-STAT pathway has been shown to activate the proliferation of ISCs (Jiang et al., 2009). Indeed, activation of a GFP reporter whose expression is controlled by ten Stat92E binding sites was obvious in all esg-mCherry-positive cells undergoing UVRAG RNAi (Fig. 4A). Accordingly, we found upregulation of cytokine expression using reporters for Unpaired1 (*upd1*) and Unpaired3 (*upd3*) (Fig. 4B,C). Interestingly, loss of UVRAG triggered expression of the Unpaired1 reporter in esg-GFP-positive cells, whereas the Unpaired3 reporter was induced in the immediate neighbors of UVRAG-deficient cells (Fig. 4B,C). These results indicate that both autocrine and niche-derived paracrine signals contribute to activation of STAT signaling in *Uvrag*-knockdown cells.

We also looked at the Jun N-terminal kinase (JNK; also known as Basket in *Drosophila*) pathway, another positive regulator of ISC proliferation in *Drosophila* (Biteau et al., 2008; Tang et al., 2013). The activity of this signaling route is very low in stem cells of wild-type guts. One of the transcriptional targets of JNK signaling is *puckered* (*puc*), a gene that encodes a phosphatase that antagonizes JNK activity. We used a *puc-lacZ* reporter line as a readout for JNK activation in the context of UVRAG loss. This transcriptional reporter is only active in enterocytes in wild-type guts (Biteau et al., 2008). In line with our data on phosphorylated JNK, the loss of UVRAG resulted in ectopic *puc-lacZ* expression in esg-GFP-positive cells (Fig. 4D). Moreover, immunolabeling using an antibody specific for the active phosphorylated form of JNK revealed a striking upregulation of phosphorylated JNK signal in all esg-GFP-positive cells undergoing UVRAG RNAi (Fig. S4C).

### JNK and STAT signaling are required to sustain hyperproliferation induced by the loss of UVRAG

**Fig. 5.**
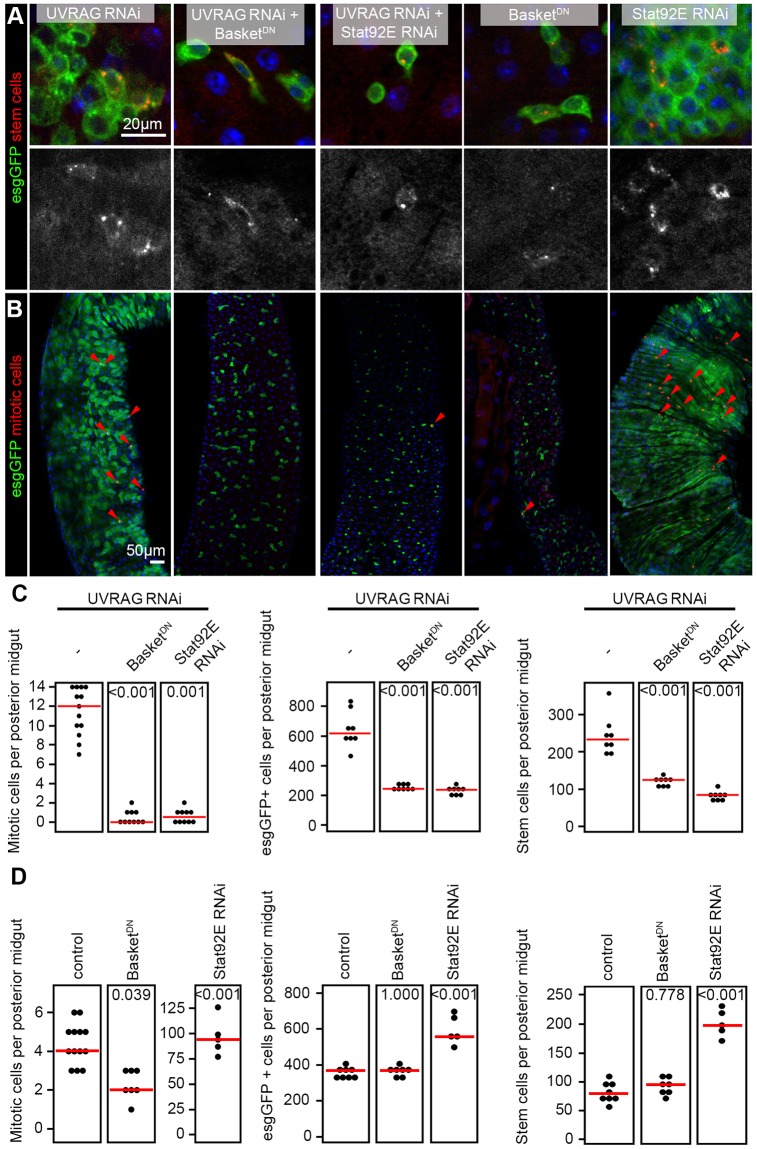
**Inhibition of JNK or STAT signaling suppresses hyperplasia in UVRAG-deficient cells.** (A) UVRAG RNAi in esg-GFP-positive cells leads to accumulation of Delta-positive ISCs and Delta-negative EBs. Inhibition of JNK or STAT signaling by expressing the dominant-negative form of Basket (JNK) or RNAi against *Stat92E* suppresses dysplasia induced by loss of UVRAG. In control experiments, the expression of dominant-negative Basket does not affect the number of esg-GFP-positive cells compared to that in wild-type guts (shown in Fig. 1B), whereas Stat92E RNAi leads to the accumulation of stem and progenitor cells. (B) Inhibition of JNK or STAT signaling blocks the increased mitotic index of UVRAG-deficient posterior midguts. In control experiments, the expression of dominant-negative Basket in esg-GFP-positive cells inhibits stem cell proliferation compared to levels found in wild-type guts (shown in Fig. 1D), whereas Stat92E RNAi leads to a massively increased mitotic index. (C) Statistical analysis of mitotic cells (shown in panel B), esg-GFP-positive stem and/or progenitor cells and Delta-positive ISCs (shown in panel A) in UVRAG RNAi, UVRAG-RNAi-positive Bsk^DN^ and UVRAG-RNAi-positive Stat92E-RNAi-expressing posterior midguts. (D) Quantification of mitotic cells (shown in panel B and Fig. 1D), esg-GFP-positive cells and ISCs (shown in panel A and in Fig. 1B) in wild-type and Bsk^DN^- and Stat92E-RNAi-expressing posterior midguts. The same control data is shown as in Fig. 1D because these experiments were carried out in parallel. In C and D, the red lines indicate median.

We sought to establish the relevance of sustained activation of these signaling pathways during hyperproliferation induced by UVRAG loss by performing epistasis analyses. Expression of a dominant-negative form of Basket (the *Drosophila* homolog of JNK) in esg-GFP-positive cells undergoing UVRAG RNAi suppressed hyperproliferation and completely restored the number of ISCs and EBs to wild-type levels (Fig. 5A-C). The knockdown of *Stat92E* also suppressed the increased mitotic activity and decreased the number of esg-GFP-positive cells in UVRAG-deficient guts (Fig. 5A-C).

In control experiments, the expression of dominant-negative Basket did not affect the number of esg-GFP-positive cells and ISCs in 3-week-old animals, although it did reduce the number of mitotic cells at this time point (Fig. 5A,B,D). RNAi knockdown of *Stat92E* increased the number of ISCs and EBs in 3-week-old animals and also led to the accumulation of mitotic cells (Fig. 5A,B,D), in line with previous reports that loss of STAT function leads to the accumulation of progenitors as early as 5-8 days owing to a block of differentiation (Beebe et al., 2010; Jiang et al., 2009).

In contrast with these results, blocking Wingless signaling through the expression of a dominant-negative version of the downstream TCF transcription factor eliminated most esg-GFP-positive cells in UVRAG-deficient and also wild-type cells (Fig. S5A,B,E-G), indicating that the Wingless pathway is required for the maintenance of ISCs regardless of the genetic background.

We sought to test whether the above pathways are specifically involved in the dysplasia of UVRAG-deficient ISCs. We thus quantified the number of esg-GFP-positive cells using genetic backgrounds that were heterozygous for STAT, JNK and Wingless components. No statistically significant differences in the number of mitoses were observed in animals heterozygous for *Wingless*, *Stat92E* (STAT) or *Basket* (JNK) (Fig. S6). In contrast, heterozygosity for *Stat92E* or *Basket*, but not *Wingless*, suppressed the hyperproliferation of UVRAG-deficient cells (Fig. S6). These data indicate that STAT and JNK signaling are specifically required to sustain UVRAG-deficiency-induced intestinal dysplasia.

### Activation of Notch promotes differentiation of UVRAG loss-of-function cells

Deregulation of Notch signaling has been found to cause a male genitalia rotation defect in hypomorphic mutant *Uvrag* animals because Notch RNAi rescues this mutant phenotype (Lee et al., 2011). Notch is a major regulator of ISC fate in *Drosophila*: its inhibition causes stem cell tumors, whereas ectopic Notch activity promotes the differentiation of ISCs into enterocytes (Micchelli and Perrimon, 2006). We thus tested whether the dysplasia of UVRAG-deficient guts could be rescued by manipulating Notch. Simultaneous knockdown of *Notch* and *Uvrag* resulted in a striking accumulation of ISCs (Fig. S7A), instead of rescuing the dysplastic phenotype. Interestingly, overexpression of the active fragment of Notch could still induce the differentiation of UVRAG-deficient ISCs and EBs to enterocytes (Fig. S7B).

### UVRAG-loss-induced dysplasia is independent of autophagy in the gut

UVRAG is famous for its dual roles in autophagy as it has been implicated both in autophagosome formation as a Vps34 kinase complex subunit and in fusion with lysosomes because it binds to the HOPS tethering complex (Liang et al., 2006, 2008). We thus tested whether its tumor suppressor function is associated with impaired autophagy in the gut.

**Fig. 6.**
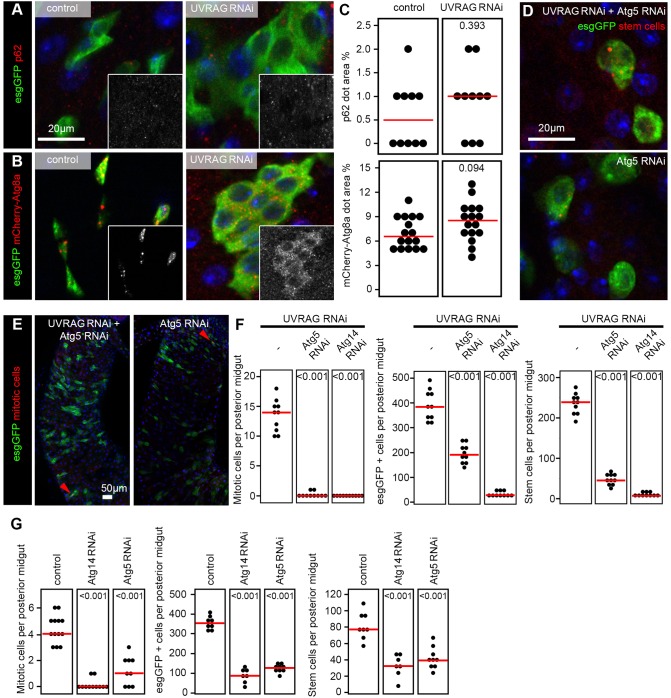
**UVRAG is dispensable for autophagy in the intestine.** (A) The levels of the selective autophagy cargo p62/Ref(2)P are similar in esg-GFP-positive cells of control and *Uvrag*-knockdown animals. (B) Structures positive for the autophagosome and autolysosome marker mCherry-tagged Atg8a form both in control and UVRAG RNAi stem and progenitor cells. Insets in A,B show the red signals in grayscale. (C) Quantification of data shown in panels A and B shows no statistically significant difference between control and UVRAG RNAi cells in the relative area of p62 aggregates or mCherry-Atg8a structures, respectively. (D) Knockdown of *Atg5* prevents UVRAG-RNAi-induced dysplasia, but expression of Atg5 RNAi alone in esg-GFP-positive cells also leads to elimination of stem and progenitor cells. (E) Atg5 depletion suppresses the UVRAG-RNAi-induced hyperproliferation response, but expression of Atg5 RNAi alone in esg-GFP-positive cells also causes a strong reduction in the number of mitotic cells. Arrowheads point to mitotic cells. (F) Quantification of data shown in panels D,E, and in A,B (UVRAG RNAi controls) and Fig. S5D shows that both Atg5 and Atg14 are required for UVRAG-loss-induced hyperproliferation and increased esg-GFP-positive and stem cell numbers. (G) Quantification of control experiments shown in panels D,E, in Fig. 1B,D and Fig. S5C shows that both Atg5 and Atg14 are required for the maintenance of stem and progenitor cells, and for mitotic activity. Red lines in C,F,G represent median.

An established test for autophagic degradation (flux) is to measure the level of the selective cargo p62, which is also known as Ref(2)P in *Drosophila* (Nezis et al., 2008; Pircs et al., 2012). No statistically significant difference was detected in endogenous p62 levels between esg-GFP-positive wild-type and UVRAG RNAi cells (Fig. 6A,C). Similarly, the direct visualization of autophagosomes and autolysosomes using mCherry-tagged Atg8a, which labels both structures (Mauvezin et al., 2014; Nagy et al., 2015), did not show a statistically significant difference between control and UVRAG-deficient esg-GFP-positive cells (Fig. 6B,C).

Knocking down the core autophagy genes *Atg5* or *Atg14* suppressed the dysplasia induced by UVRAG loss (Fig. 6D-F; Fig. S5D). However, RNAi against Atg5 or Atg14 also led to loss of esg-GFP-positive cells and decreased the number of mitoses (Fig. 6D,E,G; Fig. S5C), suggesting that autophagy is required for ISC maintenance in general, instead of for the promotion of dysplasia specifically in the absence of UVRAG. Similarly, autophagy has recently been shown to maintain muscle stem cell function and survival in mice (García-Prat et al., 2016).

### Inhibition of endocytic degradation leads to intestinal dysplasia

**Fig. 7.**
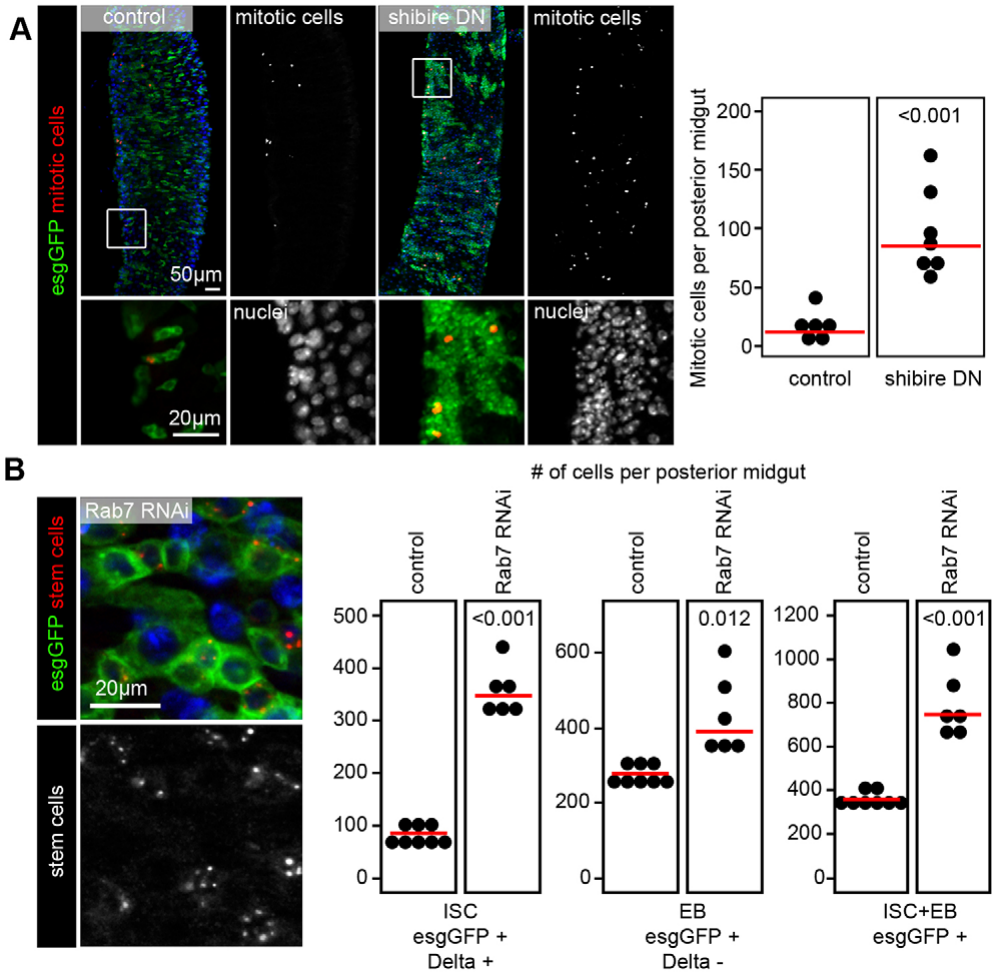
**Inhibition of endocytosis leads to intestinal dysplasia.** (A) Expression of dominant-negative shibire (dynamin) leads to a striking upregulation of mitotic activity and large-scale accumulation of small diploid esg-GFP-positive stem cells in 7-day-old animals. Boxed areas are shown enlarged in the lower panels. (B) Knockdown of *Rab7* in esg-GFP-positive cells leads to intestinal dysplasia in 3-week-old animals that is indistinguishable from that seen in UVRAG RNAi animals of a similar age. Note that the analysis of ISCs and EBs in Rab7 loss-of-function posterior midguts is compared to the same control animals as shown in Fig. 1A as experiments were performed in parallel.

Our data suggested that it is not the autophagic but the endocytic function of UVRAG that is important for preventing intestinal dysplasia. We thus analyzed the consequences of disrupting endocytosis in stem cells by expressing a dominant-negative version of shibire (shi^DN^), the *Drosophila* homolog of dynamin, which is a routinely used tool to block the endocytic downregulation of plasma membrane receptors (Moline et al., 1999). Indeed, shi^DN^ expression in esg-GFP-positive cells led to a striking enhancement of mitotic activity and a major expansion of ISCs (Fig. 7A).

The organismal effects of inhibiting dynamin-dependent endocytosis in ISCs were much more severe than those seen upon UVRAG loss as these animals died at ages of 10 to 14 days. We thus tested another well-known endocytic regulator, Rab7, which acts downstream of shibire/dynamin and promotes endosome maturation and cargo degradation (Platta and Stenmark, 2011), similar to UVRAG. Importantly, knockdown of *Rab7* in esg-GFP-positive cells perfectly phenocopied the loss of UVRAG, based on the increased number of ISCs and EBs seen in the midguts of 3-week-old animals (Fig. 7B).

## DISCUSSION

*UVRAG* encodes a homolog of yeast Vps38 in metazoans. UVRAG/Vps38 and Atg14 are mutually exclusive subunits of two different Vps34 lipid kinase complexes, both of which contain Vps34, Vps15 and Atg6/Beclin 1 (Itakura et al., 2008). It is well established that Vps38 is required for endosome maturation and vacuolar and lysosomal protein sorting, whereas Atg14 is specific for autophagy in yeast (Kihara et al., 2001). However, the function of UVRAG is much more controversial in mammalian cells. Although UVRAG was originally found to have dual roles in autophagy through promotion of autophagosome formation and fusion with lysosomes in various cultured cell lines based on, predominantly, overexpression experiments (Liang et al., 2006, 2008), recent reports have described that autophagosomes are normally generated and fused with lysosomes in the absence of UVRAG in cultured mammalian (HeLa) cells and in the *Drosophila* fat body (Jiang et al., 2014; Takáts et al., 2014).

The discoveries of *UVRAG* mutations in colorectal cancer cells, and that its overexpression increases autophagy and suppresses the proliferation of certain cancer cell lines, altogether suggest that this gene functions as an autophagic tumor suppressor (Ionov et al., 2004; Liang et al., 2006). Such a role for UVRAG is thought to be related to its binding to Beclin 1, a haploinsufficient tumor suppressor gene required for autophagy (Qu et al., 2003; Yue et al., 2003). UVRAG appears to play roles similar to yeast Vps38 in the *Drosophila* fat body, and developing eye and wing: its loss leads to the accumulation of multiple endocytic receptors and ligands in an endosomal compartment, impaired trafficking of Lamp1 and Cathepsin L to the lysosome, and defects in the biogenesis of lysosome-related pigment granules (Lee et al., 2011; Lőrincz et al., 2014; Takáts et al., 2014). However, whether this gene is also required for the maintenance of intestinal homeostasis in *Drosophila* was unclear because the loss of UVRAG did not lead to uncontrolled cell proliferation in the developing eye or wing according to these reports. Our results showing that *Uvrag* deficiency causes intestinal dysplasia suggest that this gene is also important for the proper functioning of the adult gut in *Drosophila*.

A surprising aspect of our work is that UVRAG appears to function independently of autophagy in the intestine. There are other lines of evidence that also support that UVRAG has a more important role in endocytic maturation than in autophagy. First, it has been shown that truncating mutations in *UVRAG* that are associated with microsatellite-unstable colon cancer cell lines do not disrupt autophagy (Knaevelsrud et al., 2010). Second, UVRAG depletion in HeLa cells does not prevent the formation or fusion of autophagosomes with lysosomes, but it does interfere with Egfr degradation (Jiang et al., 2014). Third, a very recent paper has shown that overexpression of the colorectal-cancer-associated truncated form of UVRAG promotes tumorigenesis independently of autophagy status, that is, both in control and Atg5-knockout cells (He et al., 2015). That paper, again, relied on the overexpression of full-length or truncated forms of UVRAG, rather than the analysis of cancer-related mutations of the endogenous locus. Fourth, the endocytic function of UVRAG has been found to be required for developmental axon pruning that is independent of autophagy in *Drosophila* (Issman-Zecharya and Schuldiner, 2014).

**Fig. 8.**
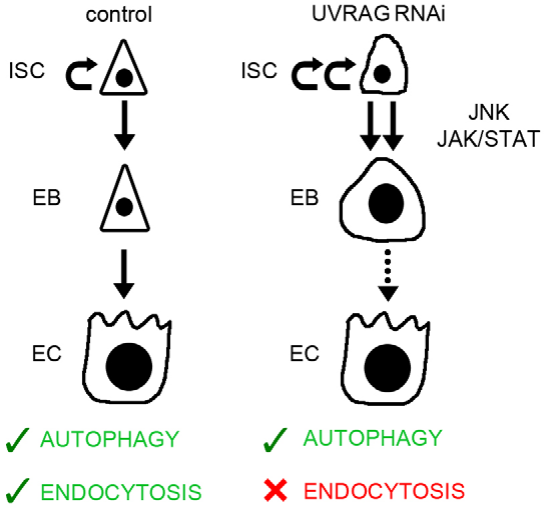
**A summary of changes in stem and progenitor cells upon loss of UVRAG.** UVRAG deficiency causes increased proliferation of ISCs through sustained activation of JNK and JAK-STAT signaling, as well as differentiation defects based on the accumulation of enlarged polyploid EBs. These alterations are likely to be due to impaired endocytic degradation, as autophagy is sustained in the absence of UVRAG.

Our results indicate that UVRAG loss is accompanied with the sustained activation of JNK and STAT signaling in ISCs and EBs, and that these pathways are required for dysplasia in this setting. Sustained activation of these signaling routes is likely to be connected to the disruption of endocytic flux in the absence of UVRAG, because inhibiting endocytic uptake or degradation through dominant-negative dynamin expression or RNAi of *Rab7*, respectively, also leads to intestinal dysplasia (Fig. 8). It is worth noting that the effects of inhibiting Shibire/dynamin function led to a much more severe hyperproliferation of ISCs and early death of animals. In line with this, the loss of early endocytic regulators, such as Rab5, in the developing eye causes overproliferation of cells and lethality during metamorphosis (Lu and Bilder, 2005). Although eye development is not perturbed by the loss of the late endocytic regulators UVRAG or Rab7 (Cherry et al., 2013; Takáts et al., 2014), these proteins are clearly important for controlling ISC proliferation and differentiation.

A recent paper shows that hundreds of RNAi lines cause the expansion of the esg-GFP compartment in 1-week-old animals (Zeng et al., 2015), which might be due to an unspecific ISC stress response in some cases. However, several lines of evidence support that impaired UVRAG-dependent endocytic degradation is specifically required to prevent intestinal dysplasia. First of all, activation of JNK stress signaling in esg-GFP-positive cells induces short-term ISC proliferation (Biteau et al., 2008), and almost all stem cells are lost through apoptosis by the 2- to 3-week age (our unpublished data), the time when the *Uvrag*-mutant phenotype becomes obvious. In fact, UVRAG loss resembles an early-onset age-associated dysplasia that is normally observed in old (30-60 days) flies and involves the simultaneous activation of both JNK and STAT signaling (Biteau et al., 2008; Osman et al., 2012). Second, UVRAG RNAi in ISCs and EBs leads to paracrine activation of the cytokine Unpaired3 in enterocytes, one of the hallmarks of niche appropriation by Notch-negative tumors (Patel et al., 2015). However, autocrine expression of the Unpaired proteins and JNK activation is observed in *Uvrag*-knockdown cells, unlike in *Notch*-negative tumors, and EBs with active Notch signaling accumulate in the absence of UVRAG, so the two phenotypes are clearly different. Third, it is the loss of autophagy that could be expected to mimic a stress response and perhaps induce stem cell tumors, but this does not seem to be the case – ISCs with Atg5 or Atg14 RNAi proliferate less in 3-week-old animals and an overall decrease of the esg-GFP compartment is seen, as opposed to the *Uvrag*-deletion phenotype.

Taken together, our work indicates that endocytic maturation and degradation serves to prevent early-onset intestinal dysplasia in *Drosophila*, and its deregulation could be relevant for the development of colorectal cancer in humans.

## MATERIALS AND METHODS

### Fly husbandry

*Drosophila melanogaster* stocks and crosses were maintained on standard cornmeal-sugar-agar medium at 22°C (uninduced condition), and gene and RNAi expression was induced in stem cells and EBs by shifting vials containing eclosing adult flies to 29°C for inhibition of thermo-sensitive Gal80 activity, until guts were examined (typically 3-week-old adult flies unless stated otherwise). In all cases, the posterior midgut of female flies was examined. The stocks used in this study were: *esg-GFP [esg-Gal4, UAS-GFP, Tub-Gal80^ts^]* (kindly provided by Philip Karpowitz) (Micchelli and Perrimon, 2006), *UAS-mCherry-Atg8a*, *esg-mCherry [esg-Gal4, UAS-mCherry-CD8, Tub-Gal80^ts^]* (generated using standard recombination techniques from the GFP version), *w[1118], UAS-Atg5 RNAi [JF02703], UAS-Atg14 RNAi [108559KK]* (Pircs et al., 2012), *UAS-Rab7-GFP*, *UAS-Uvrag RNAi [HMS01357]* (Lőrincz et al., 2014), *UAS-FYVE-GFP* (Juhász et al., 2008), *UAS-N RNAi* and *UAS-N^ICD^* (kindly provided by Heinrich Jasper) (Micchelli and Perrimon, 2006), *NRE-GFP* (Stempfle et al., 2010), *Uvrag[B21]* on *FRT40A*, *UAS-Uvrag* (Lee et al., 2011), *Uvrag[LL03097]* on *FRT40A* (Issman-Zecharya and Schuldiner, 2014), *UAS-Rab7 RNAi [40337GD]*, *UAS-shi^K44A^* (Moline et al., 1999) and *hsFLP*, *tub-Gal4*, *UAS-GFPnls; tub-Gal80*, *FRT40A* for generating mitotic clones as described previously (Biteau et al., 2010). Feeding and defecation assays were performed as described previously (Amcheslavsky et al., 2014). For epistasis analyses, we generated a stable stock of the genotype *esg-Gal4*, *UAS-GFP*, *Tub-Gal80^ts^; UAS-Uvrag RNAi [HMS01357]*, which was simply crossed to *UAS-Basket^DN^*, *UAS-Stat92E RNAi [JF01265]*, and *UAS-TCF*^Δ*N*^ to inhibit JNK, JAK-STAT and Wingless signaling, respectively. We used the JNK and cytokine reporters *Puc^E69^* (*puc-lacZ*), *upd3-lacZ* and *upd1-lacZ* (kindly provided by Bruce Edgar, German Cancer Research Center, Heidelberg, Germany), and the mutant alleles *wg^1-12^*, *wg^67c23^*, *wg^1-8^*, *stat92e^06346^*, *stat92e^HJ^*, *bsk^1^* and *bsk^2^* (all obtained from the Bloomington *Drosophila* Stock Center).

### Immunofluorescence, histology and microscopy

Isolated guts were fixed in 4% paraformaldehyde solution in PBST (PBS with 0.5% Triton X-100) for 1 h at room temperature. After extensive washes in PBST, samples were blocked in 3% BSA PBST for 2 h and incubated overnight with primary antibodies in 1% BSA PBST at 4°C. Samples were labeled with secondary antibodies in 1% BSA PBST at room temperature for 2 h. Samples were also washed after each antibody labeling step with PBST containing 4% NaCl to reduce non-specific background labeling. Fluorescent microscopy was performed as described previously (Nagy et al., 2013; Takáts et al., 2013). Primary antibodies used in this study were rat anti-mCherry (1:300) (Takáts et al., 2013), rabbit anti-p62 (1:2000) (Pircs et al., 2012), chicken anti-GFP (1:1500, Invitrogen A-10262), mouse anti-Delta (1:100, DSHB C594.9B), rabbit anti-phospho-histone H3 (1:300, Millipore 06-570), mouse anti-Prospero (1:100, DSHB MR1A), mouse anti-Wingless (1:50, DSHB 4D4), mouse anti-LacZ (1:100, DSHB 40-1a), rabbit anti-phospho-4EBP 2855 (1:200, Cell Signaling) and rabbit anti-phospho-Akt 9271, anti-phospho-JNK 9251 and anti-phospho-MAPK 9101 antibodies (all 1:50, Cell Signaling), and secondary antibodies were Alexa Fluor 488 anti-chicken (A11039), Alexa Fluor 568 anti-rabbit (A11036) and Alexa Fluor 568 anti-mouse (A11031) (all 1:1500; Life Technologies). All staining protocols were repeated at least once, with similar results.

### DSS treatment and infections

At 10 days old, flies were transferred to empty vials containing 2.5×3.5 cm filter papers soaked in 800 μl of 5% sucrose solution or 3% DSS dissolved in 5% sucrose solution for 2 days. Flies were transferred to fresh medium after treatment twice a week, and their survival was monitored. Stem cell proliferation was assessed 1 day after treatment. Infection with *Pseudomonas* was performed as described previously (Jiang et al., 2009).

### Statistical analysis

All analyses were performed in SPSS Statistics 21 software (IBM). No data or animals were excluded from the analyses. After checking the normality of data obtained in each experiment, the appropriate statistical test was applied to calculate significance values between data sets, as described previously (Nagy et al., 2013; Takáts et al., 2013). The following statistical tests were used to calculate *P*-values: two-tailed two-sample unequal variance *t*-test (Fig. 1B-D; Fig. 2B, stem cell; Fig. 3A; Fig. 7A,B; Fig. S1D,E,G; Fig. S3A), Mann–Whitney *U*-test (Fig. 1A; Fig. 2B, EBs; Fig. 5D, mitoses; Fig. 6C; Fig. S1A,B), log-rank Mantel–Cox (Fig. 3B-D), Kruskal–Wallis (Fig. 1E; Fig. 5C, mitoses; Fig. 6F,G, mitoses; Fig. S3B; Fig. S5E) and ANOVA (Fig. 5C,D, esg-GFP-positive and Delta-positive cells; Fig. 6F,G, esg-GFP-positive and Delta-positive cells; Fig. S5F,G; Fig. S6B). Hochberg and Bonferroni post-hoc tests were performed for multiple comparisons to reduce type-II errors in ANOVA and Kruskal–Wallis analyses, respectively.

## References

[DMM023416C1] Amcheslavsky A., Jiang J. and Ip Y. T. (2009). Tissue damage-induced intestinal stem cell division in Drosophila. *Cell Stem Cell* 4, 49-61. 10.1016/j.stem.2008.10.016 19128792 PMC2659574

[DMM023416C2] Amcheslavsky A., Song W., Li Q., Nie Y., Bragatto I., Ferrandon D., Perrimon N. and Ip Y. T. (2014). Enteroendocrine cells support intestinal stem-cell-mediated homeostasis in Drosophila. *Cell Rep.* 9, 32-39. 10.1016/j.celrep.2014.08.052 25263551 PMC4198943

[DMM023416C3] Beebe K., Lee W.-C. and Micchelli C. A. (2010). JAK/STAT signaling coordinates stem cell proliferation and multilineage differentiation in the Drosophila intestinal stem cell lineage. *Dev. Biol.* 338, 28-37. 10.1016/j.ydbio.2009.10.045 19896937

[DMM023416C4] Biteau B., Hochmuth C. E. and Jasper H. (2008). JNK activity in somatic stem cells causes loss of tissue homeostasis in the aging Drosophila gut. *Cell Stem Cell* 3, 442-455. 10.1016/j.stem.2008.07.024 18940735 PMC3225008

[DMM023416C5] Biteau B., Karpac J., Supoyo S., DeGennaro M., Lehmann R. and Jasper H. (2010). Lifespan extension by preserving proliferative homeostasis in Drosophila. *PLOS Genet.* 6, e1001159 10.1371/journal.pgen.1001159 20976250 PMC2954830

[DMM023416C6] Cherry S., Jin E. J., Özel M. N., Lu Z., Agi E., Wang D., Jung W.-H., Epstein D., Meinertzhagen I. A., Chan C.-C. et al. (2013). Charcot-Marie-Tooth 2B mutations in rab7 cause dosage-dependent neurodegeneration due to partial loss of function. *Elife* 2, e01064 10.7554/eLife.01064 24327558 PMC3857549

[DMM023416C7] Cordero J. B., Stefanatos R. K., Scopelliti A., Vidal M. and Sansom O. J. (2012). Inducible progenitor-derived Wingless regulates adult midgut regeneration in Drosophila. *EMBO J.* 31, 3901-3917. 10.1038/emboj.2012.248 22948071 PMC3463851

[DMM023416C8] García-Prat L., Martínez-Vicente M., Perdiguero E., Ortet L., Rodríguez-Ubreva J., Rebollo E., Ruiz-Bonilla V., Gutarra S., Ballestar E., Serrano A. L. et al. (2016). Autophagy maintains stemness by preventing senescence. *Nature* 529, 37-42. 10.1038/nature16187 26738589

[DMM023416C9] He S., Zhao Z., Yang Y., O'Connell D., Zhang X., Oh S., Ma B., Lee J.-H., Zhang T., Varghese B. et al. (2015). Truncating mutation in the autophagy gene UVRAG confers oncogenic properties and chemosensitivity in colorectal cancers. *Nat. Commun.* 6, 7839 10.1038/ncomms8839 26234763 PMC4526116

[DMM023416C10] Ionov Y., Nowak N., Perucho M., Markowitz S. and Cowell J. K. (2004). Manipulation of nonsense mediated decay identifies gene mutations in colon cancer Cells with microsatellite instability. *Oncogene* 23, 639-645. 10.1038/sj.onc.1207178 14737099

[DMM023416C11] Issman-Zecharya N. and Schuldiner O. (2014). The PI3K class III complex promotes axon pruning by downregulating a Ptc-derived signal via endosome-lysosomal degradation. *Dev. Cell* 31, 461-473. 10.1016/j.devcel.2014.10.013 25458013

[DMM023416C12] Itakura E., Kishi C., Inoue K. and Mizushima N. (2008). Beclin 1 forms two distinct phosphatidylinositol 3-kinase complexes with mammalian Atg14 and UVRAG. *Mol. Biol. Cell* 19, 5360-5372. 10.1091/mbc.E08-01-0080 18843052 PMC2592660

[DMM023416C13] Jiang H. and Edgar B. A. (2012). Intestinal stem cell function in Drosophila and mice. *Curr. Opin. Genet. Dev.* 22, 354-360. 10.1016/j.gde.2012.04.002 22608824 PMC3426656

[DMM023416C14] Jiang H., Patel P. H., Kohlmaier A., Grenley M. O., McEwen D. G. and Edgar B. A. (2009). Cytokine/Jak/Stat signaling mediates regeneration and homeostasis in the Drosophila midgut. *Cell* 137, 1343-1355. 10.1016/j.cell.2009.05.014 19563763 PMC2753793

[DMM023416C15] Jiang P., Nishimura T., Sakamaki Y., Itakura E., Hatta T., Natsume T. and Mizushima N. (2014). The HOPS complex mediates autophagosome-lysosome fusion through interaction with syntaxin 17. *Mol. Biol. Cell* 25, 1327-1337. 10.1091/mbc.E13-08-0447 24554770 PMC3982997

[DMM023416C16] Juhász G., Hill J. H., Yan Y., Sass M., Baehrecke E. H., Backer J. M. and Neufeld T. P. (2008). The class III PI(3)K Vps34 promotes autophagy and endocytosis but not TOR signaling in Drosophila. *J. Cell Biol.* 181, 655-666. 10.1083/jcb.200712051 18474623 PMC2386105

[DMM023416C17] Kapuria S., Karpac J., Biteau B., Hwangbo D. and Jasper H. (2012). Notch-mediated suppression of TSC2 expression regulates cell differentiation in the Drosophila intestinal stem cell lineage. *PLOS Genet.* 8, e1003045 10.1371/journal.pgen.1003045 23144631 PMC3493453

[DMM023416C18] Kihara A., Noda T., Ishihara N. and Ohsumi Y. (2001). Two distinct Vps34 phosphatidylinositol 3-kinase complexes function in autophagy and carboxypeptidase Y sorting in Saccharomyces cerevisiae. *J. Cell Biol.* 152, 519-530. 10.1083/jcb.152.3.519 11157979 PMC2196002

[DMM023416C19] Knaevelsrud H., Ahlquist T., Merok M. A., Nesbakken A., Stenmark H., Lothe R. A. and Simonsen A. (2010). UVRAG mutations associated with microsatellite unstable colon cancer do not affect autophagy. *Autophagy* 6, 863-870. 10.4161/auto.6.7.13033 20724836

[DMM023416C20] Kutateladze T. G. (2012). Molecular analysis of protein-phosphoinositide interactions. *Curr. Top. Microbiol. Immunol.* 362, 111-126. 10.1007/978-94-007-5025-8_6 23086416 PMC3938896

[DMM023416C21] Lee G., Liang C., Park G., Jang C., Jung J. U. and Chung J. (2011). UVRAG is required for organ rotation by regulating Notch endocytosis in Drosophila. *Dev. Biol.* 356, 588-597. 10.1016/j.ydbio.2011.06.024 21729695 PMC4414087

[DMM023416C22] Lemaitre B. and Miguel-Aliaga I. (2013). The digestive tract of Drosophila melanogaster. *Annu. Rev. Genet.* 47, 377-404. 10.1146/annurev-genet-111212-133343 24016187

[DMM023416C23] Liang C., Feng P., Ku B., Dotan I., Canaani D., Oh B.-H. and Jung J. U. (2006). Autophagic and tumour suppressor activity of a novel Beclin1-binding protein UVRAG. *Nat. Cell Biol.* 8, 688-698. 10.1038/ncb1426 16799551

[DMM023416C24] Liang C., Lee J.-S., Inn K.-S., Gack M. U., Li Q., Roberts E. A., Vergne I., Deretic V., Feng P., Akazawa C. et al. (2008). Beclin1-binding UVRAG targets the class C Vps complex to coordinate autophagosome maturation and endocytic trafficking. *Nat. Cell Biol.* 10, 776-787. 10.1038/ncb1740 18552835 PMC2878716

[DMM023416C25] Lin G., Xu N. and Xi R. (2008). Paracrine Wingless signalling controls self-renewal of Drosophila intestinal stem cells. *Nature* 455, 1119-1123. 10.1038/nature07329 18806781

[DMM023416C26] Lőrincz P., Lakatos Z., Maruzs T., Szatmári Z., Kis V. and Sass M. (2014). Atg6/UVRAG/Vps34-containing lipid kinase complex is required for receptor downregulation through endolysosomal degradation and epithelial polarity during Drosophila wing development. *BioMed. Res. Int.* 2014, 851349 10.1155/2014/851349 25006588 PMC4074780

[DMM023416C27] Lu H. and Bilder D. (2005). Endocytic control of epithelial polarity and proliferation in Drosophila. *Nat. Cell Biol.* 7, 1232-1239. 10.1038/ncb1324 16258546

[DMM023416C28] Mauvezin C., Ayala C., Braden C. R., Kim J. and Neufeld T. P. (2014). Assays to monitor autophagy in Drosophila. *Methods* 68, 134-139. 10.1016/j.ymeth.2014.03.014 24667416 PMC4048785

[DMM023416C29] Micchelli C. A. and Perrimon N. (2006). Evidence that stem cells reside in the adult Drosophila midgut epithelium. *Nature* 439, 475-479. 10.1038/nature04371 16340959

[DMM023416C30] Moline M. M., Southern C. and Bejsovec A. (1999). Directionality of wingless protein transport influences epidermal patterning in the Drosophila embryo. *Development* 126, 4375-4384. 10477304 10.1242/dev.126.19.4375

[DMM023416C31] Montagne C. and Gonzalez-Gaitan M. (2014). Sara endosomes and the asymmetric division of intestinal stem cells. *Development* 141, 2014-2023. 10.1242/dev.104240 24803650

[DMM023416C32] Nagy P., Varga A., Pircs K., Hegedűs K. and Juhász G. (2013). Myc-driven overgrowth requires unfolded protein response-mediated induction of autophagy and antioxidant responses in Drosophila melanogaster. *PLOS Genet.* 9, e1003664 10.1371/journal.pgen.1003664 23950728 PMC3738540

[DMM023416C33] Nagy P., Varga A., Kovács A. L., Takáts S. and Juhász G. (2015). How and why to study autophagy in Drosophila: it's more than just a garbage chute. *Methods* 75, 151-161. 10.1016/j.ymeth.2014.11.016 25481477 PMC4358840

[DMM023416C34] Nezis I. P., Simonsen A., Sagona A. P., Finley K., Gaumer S., Contamine D., Rusten T. E., Stenmark H. and Brech A. (2008). Ref(2)P, the Drosophila melanogaster homologue of mammalian p62, is required for the formation of protein aggregates in adult brain. *J. Cell Biol.* 180, 1065-1071. 10.1083/jcb.200711108 18347073 PMC2290837

[DMM023416C35] Ohlstein B. and Spradling A. (2007). Multipotent Drosophila intestinal stem cells specify daughter cell fates by differential notch signaling. *Science* 315, 988-992. 10.1126/science.1136606 17303754

[DMM023416C36] Osman D., Buchon N., Chakrabarti S., Huang Y.-T., Su W.-C., Poidevin M., Tsai Y.-C. and Lemaitre B. (2012). Autocrine and paracrine unpaired signaling regulate intestinal stem cell maintenance and division. *J. Cell Sci.* 125, 5944-5949. 10.1242/jcs.113100 23038775

[DMM023416C37] Patel P. H., Dutta D. and Edgar B. A. (2015). Niche appropriation by Drosophila intestinal stem cell tumours. *Nat. Cell Biol.* 17, 1182-1192. 10.1038/ncb3214 26237646 PMC4709566

[DMM023416C38] Pircs K., Nagy P., Varga A., Venkei Z., Erdi B., Hegedus K. and Juhasz G. (2012). Advantages and limitations of different p62-based assays for estimating autophagic activity in Drosophila. *PLoS ONE* 7, e44214 10.1371/journal.pone.0044214 22952930 PMC3432079

[DMM023416C39] Platta H. W. and Stenmark H. (2011). Endocytosis and signaling. *Curr. Opin. Cell Biol.* 23, 393-403. 10.1016/j.ceb.2011.03.008 21474295

[DMM023416C40] Qu X., Yu J., Bhagat G., Furuya N., Hibshoosh H., Troxel A., Rosen J., Eskelinen E.-L., Mizushima N., Ohsumi Y. et al. (2003). Promotion of tumorigenesis by heterozygous disruption of the beclin 1 autophagy gene. *J. Clin. Invest.* 112, 1809-1820. Epub 2003 Nov 24 10.1172/JCI20039 14638851 PMC297002

[DMM023416C41] Song Z., An L., Ye Y., Wu J., Zou Y., He L. and Zhu H. (2014). Essential role for UVRAG in autophagy and maintenance of cardiac function. *Cardiovasc. Res.* 101, 48-56. 10.1093/cvr/cvt223 24081163

[DMM023416C42] Stempfle D., Kanwar R., Loewer A., Fortini M. E. and Merdes G. (2010). In vivo reconstitution of gamma-secretase in Drosophila results in substrate specificity. *Mol. Cell. Biol.* 30, 3165-3175. 10.1128/MCB.00030-10 20421416 PMC2897587

[DMM023416C43] Takáts S., Nagy P., Varga A., Pircs K., Kárpáti M., Varga K., Kovács A. L., Hegedűs K. and Juhász G. (2013). Autophagosomal Syntaxin17-dependent lysosomal degradation maintains neuronal function in Drosophila. *J. Cell Biol.* 201, 531-539. 10.1083/jcb.201211160 23671310 PMC3653357

[DMM023416C44] Takáts S., Pircs K., Nagy P., Varga A., Karpati M., Hegedűs K., Kramer H., Kovacs A. L., Sass M. and Juhasz G. (2014). Interaction of the HOPS complex with Syntaxin 17 mediates autophagosome clearance in Drosophila. *Mol. Biol. Cell* 25, 1338-1354. 10.1091/mbc.E13-08-0449 24554766 PMC3982998

[DMM023416C45] Tang H.-W., Liao H.-M., Peng W.-H., Lin H.-R., Chen C.-H. and Chen G.-C. (2013). Atg9 interacts with dTRAF2/TRAF6 to regulate oxidative stress-induced JNK activation and autophagy induction. *Dev. Cell* 27, 489-503. 10.1016/j.devcel.2013.10.017 24268699

[DMM023416C46] Teitz T., Penner M., Eli D., Stark M., Bakhanashvili M., Naiman T. and Canaani D. (1990). Isolation by polymerase chain reaction of a cDNA whose product partially complements the ultraviolet sensitivity of xeroderma pigmentosum group C cells. *Gene* 87, 295-298. 10.1016/0378-1119(90)90316-J 2332174

[DMM023416C47] Yue Z., Jin S., Yang C., Levine A. J. and Heintz N. (2003). Beclin 1, an autophagy gene essential for early embryonic development, is a haploinsufficient tumor suppressor. *Proc. Natl. Acad. Sci. USA* 100, 15077-15082. 10.1073/pnas.2436255100 14657337 PMC299911

[DMM023416C48] Zeng X., Han L., Singh S. R., Liu H., Neumüller R. A., Yan D., Hu Y., Liu Y., Liu W., Lin X. et al. (2015). Genome-wide RNAi screen identifies networks involved in intestinal stem cell regulation in Drosophila. *Cell Rep.* 10, 1226-1238. 10.1016/j.celrep.2015.01.051 25704823 PMC4420031

[DMM023416C49] Zhao Z., Oh S., Li D., Ni D., Pirooz S. D., Lee J.-H., Yang S., Lee J.-Y., Ghozalli I., Costanzo V. et al. (2012). A dual role for UVRAG in maintaining chromosomal stability independent of autophagy. *Dev. Cell* 22, 1001-1016. 10.1016/j.devcel.2011.12.027 22542840 PMC3356442

